# Refinement of risk stratification for childhood rhabdomyosarcoma using FOXO1 fusion status in addition to established clinical outcome predictors: A report from the Children's Oncology Group

**DOI:** 10.1002/cam4.2504

**Published:** 2019-08-27

**Authors:** Emily Hibbitts, Yueh‐Yun Chi, Douglas S. Hawkins, Frederic G. Barr, Julie A. Bradley, Roshni Dasgupta, William H. Meyer, David A. Rodeberg, Erin R. Rudzinski, Sheri L. Spunt, Stephen X. Skapek, Suzanne L. Wolden, Carola A. S. Arndt

**Affiliations:** ^1^ Department of Biostatistics University of Florida Gainesville Florida; ^2^ Division of Hematology/Oncology Fred Hutchinson Cancer Research Center Seattle Children's Hospital University of Washington Seattle Washington; ^3^ Laboratory of Pathology National Cancer Institute Bethesda Maryland; ^4^ Department of Radiation Oncology University of Florida Health Science Center Jacksonville Florida; ^5^ Division of Pediatric General and Thoracic Surgery Cincinnati Children's Hospital Medical Center Cincinnati Ohio; ^6^ Department of Pediatrics University of Oklahoma Health Sciences Center Oklahoma City Oklahoma; ^7^ Department of Surgery Brody School of Medicine East Carolina University Greenville North Carolina; ^8^ Department of Laboratories Seattle Children's Hospital Seattle Washington; ^9^ Department of Pediatrics Stanford University School of Medicine Palo Alto California; ^10^ Department of Hematology and Oncology UT Southwestern Medical Center Dallas Texas; ^11^ Department of Radiation Oncology Memorial Sloan Kettering Cancer Center New York City New York; ^12^ Department of Pediatric and Adolescent Medicine Mayo Clinic Rochester Minnesota

**Keywords:** fusion status, rhabdomyosarcoma, risk stratification, survival tree regression

## Abstract

**Background:**

Previous studies of the prognostic importance of FOXO1 fusion status in patients with rhabdomyosarcoma (RMS) have had conflicting results. We re‐examined risk stratification by adding FOXO1 status to traditional clinical prognostic factors in children with localized or metastatic RMS.

**Methods:**

Data from six COG clinical trials (D9602, D9802, D9803, ARST0331, ARTS0431, ARST0531; two studies each for low‐, intermediate‐ and high‐risk patients) accruing previously untreated patients with RMS from 1997 to 2013 yielded 1727 evaluable patients. Survival tree regression for event‐free survival (EFS) was conducted to recursively select prognostic factors for branching and split. Factors included were age, FOXO1, clinical group, histology, nodal status, number of metastatic sites, primary site, sex, tumor size, and presence of metastases in bone/bone marrow, soft tissue, effusions, lung, distant lymph nodes, and other sites. Definition and outcome of the proposed risk groups were compared to existing systems and cross‐validated results.

**Results:**

The 5‐year EFS and overall survival (OS) for evaluable patients were 69% and 79%, respectively. Extent of disease (localized versus metastatic) was the first split (EFS 73% vs 30%; OS 84% vs. 42%). FOXO1 status (positive vs negative) was significant in the second split both for localized (EFS 52% vs 78%; OS 65% vs 88%) and metastatic disease (EFS 6% vs 46%; OS 19% vs 58%).

**Conclusions:**

After metastatic status, FOXO1 status is the most important prognostic factor in patients with RMS and improves risk stratification of patients with localized RMS. Our findings support incorporation of FOXO1 status in risk stratified clinical trials.

## Graphical abstract

The importance of FOXO1 fusion status in risk stratification for treatment assignment in patients with rhabdomyosarcoma (RMS) has been controversial. Survival tree regression was conducted in a group of 1727 RMS patients treated on 6 clinical trials to recursively select prognostic factors with the greatest impact on event‐free survival, and demonstrated that after metastatic status, FOXO1 fusion status is the most important prognostic factor in childhood RMS.

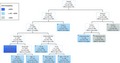

## 1 INTRODUCTION

Rhabdomyosarcomas (RMS) constitute 40% of soft tissue sarcomas in children, with an incidence of 4.5 cases per million children and adolescents per year.^1^ Risk stratification to guide therapy intensity traditionally includes clinical factors present at diagnosis. Analysis of patient and disease characteristics of patients with nonmetastatic RMS treated on the third and fourth Intergroup Rhabdomyosarcoma Studies (IRS‐III and IRS‐IV) identified prognostic significance of histology, stage, clinical group, and primary site. Subsequent clinical studies from 1997 to 2004 divided patients into two low‐risk, one intermediate‐risk, and one high‐risk prognostic subgroups for treatment assignment.^2^ Oberlin and colleagues performed a multivariate analysis of risk factors in 788 patients with metastatic RMS treated in nine studies performed by European and American cooperative groups from 1984 to 2000.^3^ Inferior event‐free survival (EFS) was correlated with age under 1 year or older than 10 years, unfavorable site of primary tumor (defined as extremity and “other” sites), presence of three or more sites of metastatic disease, and presence of bone or bone marrow involvement; histology was not independently associated with outcome.

Cytogenetic studies identify a frequent t(2;13)(q35;q14) or variant t(1;13)(p36;q14) chromosomal translocation in most cases of alveolar RMS (ARMS). These translocations involve the *PAX3* gene on chromosome 2 or the *PAX7* gene on chromosome 1 and the *FOXO1* gene on chromosome 13 to generate *PAX3‐FOXO1* or *PAX7‐FOXO1* fusion genes, which encode fusion proteins with oncogenic activity. Molecular pathologic analysis of fusion status revealed that 80% of ARMS cases contain a FOXO1 fusion (60% with *PAX3‐FOXO1* fusion and 20% with *PAX7‐FOXO1* fusion) whereas the vast majority (>95%) of embryonal RMS (ERMS) cases do not contain any FOXO1 fusion.^4^

Two retrospective studies on patients treated on multiple different clinical trials spanning two decades have shown conflicting results on the prognostic significance of FOXO1 fusion status. Williamson et al found that fusion‐positive patients have an inferior outcome compared to fusion‐negative patients, whereas Stegmaier et al showed no association between outcome and fusion status in patients with ARMS.^5^, ^6^ Analyses of patients with low‐ (n = 16) and intermediate‐risk (n = 434) RMS treated on a series of recent Children's Oncology Group (COG) trials confirmed the prognostic significance of FOXO1 fusion status.^7^, ^8^

The purpose of the current study was to determine if the childhood RMS risk stratification algorithm could be further strengthened with the addition of FOXO1 fusion status to traditional clinical features in a cohort of nearly 2000 patients from the six most recent COG clinical trials. We performed statistical modeling incorporating known risk factors, including FOXO1 fusion status, to determine which risk factors were most important in determining outcomes. Based on the results of this modeling, we propose new risk group definitions that more accurately segregate patients into meaningful prognostic subgroups than the previous risk stratification system.

## 2 MATERIALS AND METHODS

Patients with newly diagnosed RMS enrolled on six previously reported COG studies, shown in Table 1, conducted from 1997 to 2013 were included in this analysis.^9^, ^10^, ^11^, ^12^, ^13^, ^14^ These trials were approved by the institutional review boards of each participating institution or Pediatric Central Review Board, as required. Informed consent/assent from the patient and/or parent/guardian as appropriate was obtained before enrollment.

**Table 1:** Risk group assignment by risk group definition

Risk group assignment	Risk group stratification definition	
	D‐series (1997‐2004)	ARST‐series (2004‐2013)
Low	D9602 (NCT00002995): (ERMS only) **Subset A**: Fav site, any size, Stage 1, Group I and II, N_0_; Fav site, any size, Stage 1, Group III, N_0_, (orbit only); Unfav site, ≤ 5cm, Stage 2, Group I, N_0_, N_x_; Therapy: VA x 45 wk **Subset B**: Fav site, any size, Stage 1, Group II, N_1_; Fav site, any size, Stage 1, Group III, N_1_ (orbit only); Fav site (except orbit), any size, Stage 1, Group III, N_0_, N_1_; Unfav site, ≤5 cm, Stage 2, Group II, N_0_, N*_x_*; Unfav site, ≤5 cm with N_1_ or >5 cm any size, Stage 3, Group I/II, N_0_, N*_x_*, N_1_ Therapy similar to D9803	ARST0331 (NCT00075582): (ERMS only) **Subset 1**: Stage 1, Group I and II, N_0_; Stage 1, Group III, N_0_, N*_x_*, (orbit only); Stage 2, Group I, N_0_, N*_x_*, and Group II; Therapy: VAc x 4, VA x4 **Subset 2**: Stage 1, Group III, N_0_, N*_x_*, (non‐orbital); Stage 3, Group I/II Therapy VAc x 4, VA x 12
Intermediate	D9803 (NCT00003958) Stage 1‐3, Group I‐III, ARMS; Stage 2/3, Group III, ERMS; Stage 4, Group IV, ERMS, <10 years Therapy: VAC vs VAC/VTC	ARST0531 (NCT00354835) Stage 2/3, Group III, ERMS; Stage 1‐3, Group I‐III, ARMS Therapy: VAc vs VAc/VI
High	D9802 (NCT00003955) Stage 4, Group IV, except ERMS <10 y Therapy: I ± V; VAC	ARST0431 (NCT00354744) Stage 4, Group IV Therapy: VDc/IE/I/VAc

Abbreviations: ARMS, alveolar rhabdomyosarcoma; ERMS, embryonal rhabdomyosarcoma; Fav, favorable; I, irinotecan; N_0_, No regional nodal involvement; N_1_, regional nodal involvement; N*_x_*, nodal involvement unknown; Unfav, unfavorable; V, vincristine; VA, vincristine/actinomycin; VAc, vincristine, actinomycin/cyclophosphamide (dose 1.2 gm/m^2^); VAC, vincristine, actinomycin/cyclophosphamide (dose 2.2 gm/m^2^); VDc, vincristine/doxorubicin/cyclophosphamide; IE, Ifosfamide/etoposide; VTC, vincristine, topotecan, cyclophosphamide.

Prognostic factors incorporated in the model include age at diagnosis (categorized based on previous studies into <1, 1 to 9, 10+ years),^15^ sex, primary site (favorable (orbit, head and neck (excluding parameningeal), genitourinary (excluding bladder/prostate), and biliary tract/liver) versus unfavorable (bladder/prostrate, extremity, cranial parameningeal, other (includes trunk, retroperitoneum, pelvis, perineal/perianal, intrathoracic, gastrointestinal)), tumor size (≤5 cm, >5 cm), histology (ARMS, ERMS), FOXO1 fusion status (positive, negative), clinical group (I, II, III, IV),^16^ nodal status (N_0_, nodal involvement absent; N_1_, nodal involvement present), number of metastatic sites (ranging from 0 to 6), and presence or absence of metastasis in the following sites: bone or bone marrow as a single variable, distant soft tissue, pleural effusion, lung, distant lymph nodes, and other sites. Tumor invasiveness was not included as it is not used in current risk stratification. Stage was not included because all elements of stage (primary site, tumor size, regional nodal status, and presence of metastases) were individually included in the analysis. Only patients with complete data were eligible for analyses. Since virtually all ERMS are fusion‐negative, the 1270 ERMS patients without FOXO1 fusion data were assumed to be fusion‐negative.^17^, ^18^, ^19^ Within our study population, only 1 of the 58 (1.7%) ERMS patient with known fusion data were fusion‐positive. When centralized FOXO1 fusion testing (using previously reported reverse transcription—polymerase chain reaction (RT‐PCR)^20^ or fluorescence in situ hybridization (FISH)^21^ and methodology was not available, institutional assessment was used (n = 51 patients). Patients with histology other than ARMS, ERMS, botryoid or spindle cell/sclerosing RMS (n = 128) and those with missing clinical data (n = 353) were also excluded yielding 1727 evaluable patients.

Event‐free survival was the primary endpoint and was calculated from date of study enrollment to date of first event, which included tumor recurrence or progression, secondary malignancy, and death due to any cause, or date of last contact for those without events. EFS was selected as the primary endpoint for this analysis as the risk stratification system should be designed to evaluate outcomes of upfront therapy. In addition, overall survival (OS) provides fewer events than EFS, resulting in lower power to stratify subsets of patients.

We conducted survival tree regression for EFS to determine the prognostic impact of the risk factors discussed above using the R package “partykit” to produce a survival tree.^22^ We selected this method as it reduces variable selection bias towards variables with more than a single cut point. In addition, this method reduces over‐fitting by implementing a statistically motivated stopping criterion. The model stops splitting when there is no longer a statistically significant split and this eliminates the need for pruning. Recursively, the factor most strongly associated with the EFS in the univariate fashion was selected for branching and split using a goodness of split measure that optimizes between‐node separation using the log‐rank statistics.^23^, ^24^ This process continues until no statistically significant associations exist. The algorithm utilizes the log‐rank score at a 5% significance level and the Bonferroni method to adjust for multiple testing at each split. The 5‐year EFS and OS rates for each leaf in the EFS survival tree were estimated using the Kaplan‐Meier method.^24^ Confidence intervals for estimates of time‐to‐event distributions were calculated using Peto and Peto's formula.^25^

Once results of the survival tree were known, we used EFS from terminal leaves to revise the low‐, intermediate‐ and high‐risk definitions. Risk group assignment was arbitrary, but was based on cut‐offs previously derived from an analysis of patients with non‐metastatic disease enrolled on IRS‐III and IRS‐IV.^2^ Low risk was defined as having a 5‐year EFS of ≥90%. Intermediate risk was defined as having a 5‐year EFS of ≥40% to <90%. High risk was defined as having a 5‐year EFS of <40%. Cross‐validation (10‐fold) was utilized for internal validation.^26^ Concordance was calculated to assess consistency of cross‐validated risk groups with the revised risk groups from the survival tree for all evaluable patients.^27^ Risk stratifications utilized on the D‐series and ARST‐series studies (Table 1) were applied to the entire analytic cohort. This analysis allows for comparison of risk assignment and performance characteristics of risk groups based on the different risk stratification definitions. EFS curves were compared using the log‐rank test for each risk group stratification definition.^28^

## 3 RESULTS

**Figure 1:**
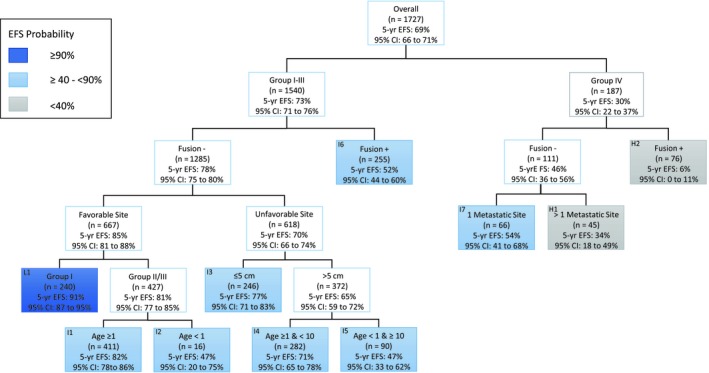
Event‐free survival (EFS) tree of analytic cohort with terminal leaves labeled by risk groups. EFS, event‐free survival; Fusion, FOXO1 fusion status

Table 2 shows characteristics for 2028 eligible patients. The estimated 5‐year EFS and OS rates for all 1727 evaluable patients with complete data were 69% (95% CI: 66%‐71%) and 79% (95% CI: 77%‐82%), respectively. The most significant risk factor in relation to EFS resulting was clinical group (Figure 1). The 5‐year EFS and OS for patients with localized disease (Group I, II, and III) were 73% and 84% compared to 30% and 42% for patients with metastatic disease (Group IV) (Figure 1; Table 3).

**Table 2:** Patient and clinical characteristics

Characteristic	No. of eligible patients	%	No. of evaluable patients	%
Total No.	2028		1727	
Study				
D9602	403	20	372	22
D9802	111	5	60	2
D9803	616	30	477	28
ARST0331	341	17	319	18
ARST0431	109	5	86	5
ARST0531	448	22	413	24
Age, y				
<1	100	5	83	5
1‐9	1241	61	1091	63
≥10	687	34	553	32
Sex				
Male	1233	61	1050	61
Female	795	39	677	39
Clinical group				
I	333	16	293	17
II	343	17	304	18
III	1081	53	943	55
IV	268	13	187	11
Missing	3	0		
Histology				
Alveolar	572	28	434	25
Embryonal	1328	65	1293	75
Missing	128	6		
Tumor size, cm				
≤5	1077	53	948	55
>5	912	45	779	45
Missing	39	2		
Regional lymph node status				
N0	1625	80	1416	82
N1	379	19	311	18
Missing	24	1		
Primary site				
Favorable	832	41	735	43
Unfavorable	1193	59	992	57
Missing	3	0		
Bone/bone marrow metastases				
No	1900	94	1648	95
Yes	127	6	79	5
Missing	1	0		
Lung metastases				
No	1912	94	1643	95
Yes	84	6	84	5
Missing	3	0		
Distant node metastases				
No	1939	96	1673	97
Yes	85	4	54	3
Missing	4	0		
Distant soft tissue metastases				
No	1969	97	1685	98
Yes	57	2	42	2
Missing	2	0		
Pleural effusion				
No	1987	98	1703	99
Yes	40	2	24	1
Missing	1	0		
Other sites metastases				
No	1918	95	1653	96
Yes	109	5	74	4
Missing	1	0		
Number of metastatic sites				
0	1751	86	1531	89
1	131	6	98	6
2	70	3	56	3
3	48	2	26	2
4	17	1	12	1
5	6	0	3	0
6	1	0	1	0
Missing	1	0		
FOXO1 fusion status^a^				
Fusion‐negative^b^	1445	71	1396	81
Fusion‐positive	351	17	331	19
Missing	232	11		

Abbreviations: N0, No regional nodal involvement; N1, Regional nodal involvement.

a FOXO1 fusion data sources included centralized testing and institutional reports of reverse transcription polymerase chain reaction, fluorescence in situ hybridization, or cytogenetics.

b ERMS patients with missing fusion status assumed Fusion‐negative.

**Table 3:** Overall survival for terminal leaves from event‐free survival tree

Risk group	Terminal leaf	Clinical group	Fusion status	Primary site	Age, years	Tumor size	Number of metastatic sites	5‐year OS, %	95% CI of 5‐year OS
Low	L1	I	Negative	Favorable	Any	Any	NA	99	97‐100
Intermediate	I1	II/III	Negative	Favorable	≥1	Any	NA	93	90‐96
	I2	II/III	Negative	Favorable	<1	Any	NA	80	59‐100
	I3	I‐III	Negative	Unfavorable	Any	≤5cm	NA	85	80‐91
	I4	I‐III	Negative	Unfavorable	≥1 or <10	>5cm	NA	81	75‐86
	I5	I‐III	Negative	Unfavorable	<1 or ≥10	>5cm	NA	61	46‐75
	I6	I‐III	Positive	Any	Any	Any	NA	65	58‐73
	I7	IV	Negative	Any	Any	Any	1	70	57‐82
High	H1	IV	Negative	Any	Any	Any	>1	40	24‐56
	H2	IV	Positive	Any	Any	Any	Any	19	10‐28

Abbreviations: CI, confidence interval; NA, not applicable; OS, overall survival.

Among patients with localized disease, FOXO1 fusion status was the strongest prognostic factor. The model did not discriminate any further risk factors among the fusion‐positive patients after fusion status. Fusion‐negative patients had a 5‐year EFS of 78% and 5‐year OS of 88%. Fusion‐positive patients had a 5‐year EFS of 52% and OS of 65%.

Among 1285 fusion‐negative patients, primary site (favorable versus unfavorable) was the strongest prognostic factor. Patients with favorable sites had a 5‐year EFS of 85% and 5‐year OS of 95% and patients with unfavorable sites had a 5‐year EFS of 70% and 5‐year OS of 80%. Within the favorable site cohort, patients in Group I had better outcomes than patients in Group II and III (5‐year EFS: 91% vs 81%; 5‐year OS: 99% vs 93%). For FOXO1 fusion‐negative patients with Group II and III tumors in favorable sites, those older than 1 year had better outcome than those less than 1 year (5‐year EFS: 82% vs 47%; 5‐year OS: 93% vs 80%).

For FOXO1 fusion‐negative patients with unfavorable sites, tumor size was the strongest prognostic factor. Patients with small tumors (≤5 cm) had a 5‐year EFS of 77% and 5‐year OS of 85% and patients with large tumors (>5 cm) had a 5‐year EFS of 65% and 5‐year OS of 76%. For patients with large tumors, those 1‐10 years old had better outcome than those under 1 year or over 10 years (5‐year EFS: 71% vs 47%; 5‐year OS: 81% vs 61%).

For patients with metastatic disease, FOXO1 fusion status was the strongest prognostic variable. Similar to patients with localized and fusion‐positive disease, no clinical factor further subdivided outcome among patients with metastatic and fusion‐positive disease. Fusion‐negative patients had a 5‐year EFS of 46% and 5‐year OS of 58%, whereas fusion‐positive patients had a 5‐year EFS of 6% and 5‐year OS of 19%. For the 111 fusion‐negative patients, the number of metastatic sites was the strongest prognostic factor. Those with a single metastatic site had better outcomes than those with more than 1 metastatic site (5‐year EFS: 54% vs 34%; 5‐year OS: 70% vs 40%).

Based on EFS cut points from the survival tree, we propose new risk groups definitions. Our revised risk definitions include a small, but well defined low‐risk group with excellent outcome, a much larger intermediate‐risk group with a wider range of outcomes, and another small, but well‐defined high‐risk group. We set the boundary for low‐risk at a 5‐year EFS of 90% or greater which included patients with clinical group I, favorable site, and FOXO1 fusion‐negative tumors. The boundaries for intermediate‐risk were 5‐year EFS greater than 40% and less than 90% and included patients with clinical group II and III, favorable site, FOXO1 fusion‐negative; clinical group I‐III FOXO1 fusion‐negative tumors in an unfavorable site; clinical group I‐III, FOXO1 fusion‐positive tumors; and clinical group IV, FOXO1 fusion‐negative tumors involving only one metastatic site. A 5‐year EFS of less than 40% marked the boundary for high‐risk which included patients with clinical group IV and FOXO1 fusion‐positive and those group IV, FOXO1 fusion‐negative with more than one metastatic site. Table 4 presents the percentage of patients and outcomes for the analytic cohort based on the D‐series, ARST‐series, and proposed risk group, and cross‐validated definitions. Concordance between cross‐validated risk groups and proposed risk groups was 1598/1727 = 92.3%.

**Table 4:** Percentage of patients and outcomes by risk group and risk group definition

Risk group definition	Risk group assignment								
	Low			Intermediate			High		
	% of patients	5‐year EFS, %	95% CI	% of patients	5‐year EFS, %	95% CI	% of patients	5‐year EFS, %	95% CI
D‐series	41.5	84	81‐87	51.4	63	59‐67	7.1	18	10‐25
ARST‐series	41.5	84	81‐87	47.6	64	60‐68	10.9	30	22‐37
Proposed	13.9	91	87‐95	79.1	69	66‐72	7.0	16	9‐23
Cross‐validated	14.2	89	85‐94	81.0	68	65‐71	4.8	16	8‐25

Abbreviations: CI, confidence interval; EFS, event‐free survival.

**Figure 2:**
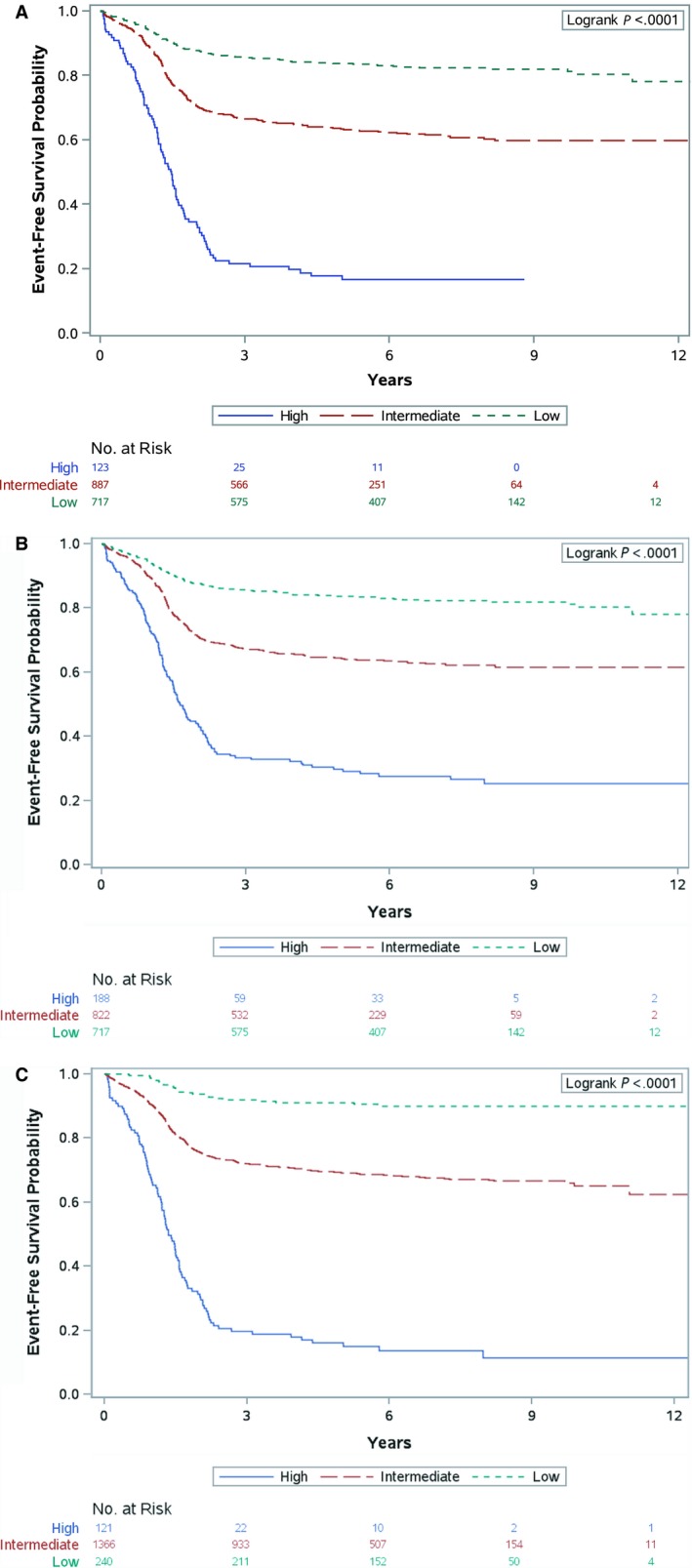
EFS curves by risk group as defined by (A) D‐series criteria; (B) ARST‐series criteria; (C) proposed stratification

Our proposed risk stratification of low‐, intermediate‐ and high‐risk changed the percentages of patients in each category. A higher percentage of patients were assigned to low‐risk by D‐series and ARST‐series definitions than by the proposed risk stratification. The 5‐year EFS for patients in the proposed stratification for low‐risk (91%) was higher than those in the D‐series (84%) and ARST‐series (84%). A lower percentage of patients in past studies were assigned to intermediate‐risk than by the proposed stratification. The 5‐year EFS for patients in the proposed stratification for intermediate‐risk (69%) was higher than those in the D‐series (63%) and ARST‐series (64%). The percentage of high‐risk patients in the proposed stratification was similar to that of the D‐series and lower than the ARST‐series. The 5‐year EFS for high‐risk patients in the proposed stratification (16%) is lower than those in the D‐series (18%) and ARST‐series (30%). Kaplan‐Meier plots for the three risk stratification definitions are presented in Figure 2A‐C.

## 4 DISCUSSION

In this large cohort of contemporarily treated patients with RMS, incorporating FOXO1 fusion status into the risk stratification algorithm separated out a small group of patients with an excellent 5‐year EFS of 91%, characterized by favorable site, FOXO1 fusion‐negative status, and clinical group I disease. These patients all received either two drug therapy with VA for 45 weeks or three drug therapy with 4 cycles of VAC and four cycles of VA. The excellent outcome and small number of patients in this group (14% of the patients analyzed) will make future studies to further refine therapy challenging.

We separated the remaining patients into two groups: an expanded intermediate‐risk group with a 5 year EFS ranging from 47% to 82%, (79% of the total patients analyzed) and a high‐risk group with a 5 year EFS of 6%‐34% (7% of the patients analyzed). Table 4 shows the characteristics of the newly defined risk groups based on this analysis.

Although the range of 5‐year EFS is large in the intermediate‐risk group, these patients would lend themselves to future prospective study of therapeutic strategies and molecular features associated with outcome. For example, MG5, a 5 gene metagene signature is strongly associated with outcome in two separate cohorts of FOXO1 fusion‐negative patients with RMS.^29^, ^30^ In an analysis of D9803 intermediate‐risk patients, low MG5 score had an EFS similar to the low‐risk patients described above.^29^ If the prognostic value of the MG5 score can be confirmed in a larger prospective study which also includes fusion‐positive patients, it could be used to identify additional patients warranting less intensive therapy. Although patients with low MG5 scores had an excellent outcome, those treated on D9803 received intensive 3‐4 drug therapy. If these patients could be identified prospectively, they could be targeted for therapy reduction. Similarly, patients with a high MG5 score had a very poor outcome similar to the FOXO1 fusion‐positive patients, and could be targeted for novel approaches used for patients with metastatic disease.

Our model identified a more favorable subset of patients with metastatic disease: FOXO1 fusion‐negative patients with only one site of metastasis, similar to the clinical findings of Oberlin et al.^3^ In this more favorable subset, 48 of 66 patients were under 10 years at diagnosis. Modifications to the risk stratification algorithm moved young patients with metastatic ERMS from intermediate‐risk on the D‐series of studies to high‐risk on the ARST‐series of studies. In contrast to an earlier analysis of a larger cohort of patients with metastatic RMS that did not include FOXO1 fusion status and found that age 10 years and greater predicted a less favorable outcome, our analysis only identified the presence of more than one metastatic site as a predictor of worse outcome (and only for FOXO1 fusion‐negative tumors).^3^ In an analysis of fusion status and outcome on two high‐risk RMS studies (D9802 and ARST0431), Rudzinski et al found Oberlin score to be more prognostic than fusion status but did not include metastatic patients from D9803.^31^ Although the high‐risk group proposed here comprises few patients, their poor outcome (EFS 6%‐34%) lends itself to higher risk investigational studies to identify more effective therapies.

With the new risk stratification algorithm using this survival tree, the low‐ and high‐risk groups become smaller, and the intermediate‐risk group expands. The EFS dividing line between risk groups is arbitrary, but is intended to allow modulation of treatment intensity within clinical trials based upon the risk of disease recurrence. The intermediate risk and high risk group outcomes are still unsatisfactory, and justify exploration of innovative new therapeutic approaches with the goals to improve outcomes and define new treatment alternatives.

There are several differences between our analysis and that used previously to define IRS/COG risk stratification in localized RMS, limiting comparability of the two analyses.^2^ The prior IRS/COG analysis did not have FOXO1 fusion status available to include in the model, and excluded patients with metastatic disease and included patients with undifferentiated sarcoma, whereas we did the reverse.

One potential limitation in our analysis was that patients with ERMS were assumed to be FOXO1 fusion‐negative for analytic purposes as few had undergone FOXO1 fusion testing. Since cases classified as ERMS by expert central pathology review are rarely FOXO1 fusion‐positive, FOXO1 fusion testing likely would not have altered the stratification of these patients.^17^, ^18^, ^19^ Other potential flaws in our study design include the use of both RT‐PCR and FISH for FOXO1 fusion detection and incorporation of institutional FOXO1 fusion assessment when centralized testing was not available. In addition, the much rarer fusions in rhabdomyosarcoma (such as PAX‐NCOA, which are not detectable by FOXO1 FISH or PCR studies) were not looked for, and it is not known at this time how they affect outcome.^32^ In addition, we only considered pretreatment factors and not therapy received. Moreover, because none of the randomized studies showed any difference between treatment regimens, treatment was not considered as an independent variable.

In conclusion, after metastatic status, FOXO1 fusion status is the most important prognostic factor in patients with RMS. The current COG intermediate‐risk study, ARST1431, allocates treatment based on FOXO1 fusion testing. Since prognosis depends in part on treatment, our results are only applicable to patients treated on COG studies until they have been confirmed using an independent data set, for example from patients treated on the European Paediatric Soft Tissue Sarcoma RMS 2005 study with a different chemotherapy backbone and local control philosophy. The current effort to create an international dataset of childhood soft tissue sarcomas through combined efforts of a number of international cooperative groups may make further analyses to confirm these results feasible.

## CONFLICT OF INTEREST

None: Dasgupta, Chi, Meyer, Rudzinski, Hibbitts, Rodeberg, Skapek, Spunt. Positive disclosures: Arndt ‐‐ stock ownership Pfizer and Merck; Bradley‐‐ honoraria and travel funding from Ion Beam applications; Hawkins‐‐ travel funding from Bayer, BMS, loxo oncology, Celgene; Barr‐‐ stock ownership by self or immediate family member in Abbott Labs, General Electric, Danaher corp, Abbvie inc, Baxter international, Celgene, Colgate Palmolive, Edwards lifesciences, Eli Lilly, Johnson and Johnson; Wolden—travel expenses from IBA and consulting role in Y‐mAbs.

## AUTHOR CONTRIBUTIONS

All authors have contributed toward the article in the following ways: (a) substantial contributions to the conception and design or analysis and interpretation of the data; (b) drafting the article or revising it critically for important intellectual content; (c) final approval of the version to be published; and (d) agreement to be accountable for all aspects of the work.

Precis: The importance of FOXO1 fusion status in risk stratification for treatment assignment in patients with rhabdomyosarcoma (RMS) has been controversial. Survival tree regression was conducted in a group of 1727 RMS patients treated on 6 clinical trials to recursively select prognostic factors with the greatest impact on event‐free survival (EFS), and demonstrated that after metastatic status, FOXO1 fusion status is the most important prognostic factor in childhood RMS.
