# Supplementary material for: Refinement of risk stratification for childhood rhabdomyosarcoma using FOXO1 fusion status in addition to established clinical outcome predictors: A report from the Children's Oncology Group
Source: Cancer Med. 2019 Aug 27;8(14):6437–48. doi: 10.1002/cam4.2504 (PMC6797586; doi:10.1002/cam4.2504)
Supplement: Supplementary file 2 [file CAM4-8-6437-s002.docx]

Supplemental

Cross-validation Procedure:

1. Randomly split data from evaluable patients into 10 roughly equal folds.
2. For the first fold, run survival tree regression using partykit to produce a survival tree.
3. Determine the 5-year EFS for each terminal node in the survival tree.
4. Based on EFS, determine the cross-validation risk group assignment (low: ≥ 90, intermediate: ≥ 40 to < 90, high: <40) for each terminal node in the survival tree.
5. Repeat this procedure for the remaining 9 folds.
6. Once all patients have a cross-validation risk group assignment, compare the risk group assigned in cross-validation to the risk group assigned in the full analysis and calculate concordance.

Reliability of the nodes from cross-validation:

Group (I-III vs IV) was chosen as the first split for all 10 cross-validation trees.

For the first branch in the tree, Group I-III was selected for all 10 trees, fusion - was chosen for 7 of the trees, favorable site was chosen for 4 of the trees, and Group I was chosen for 2 of the trees.

For the second branch in the tree, Group I-III was selected for all 10 trees, fusion - was chosen for 7 of the trees, favorable site was chosen for 4 of the trees, Group II/III was chosen for 2 of the trees, and age ≥ 1 was chosen for 1 of the trees.

For the third branch in the tree, Group I-III was selected for all 10 trees, fusion - was chosen for 7 of the trees, favorable site was chosen for 4 of the trees, Group II/III was chosen for 2 of the trees, and age < 1 was chosen for 1 of the trees.

For the fourth branch in the tree, Group I-III was selected for all 10 trees, fusion - was chosen for 7 of the trees, unfavorable site was chosen for 4 of the trees, and tumor size ≤ 5cm was chosen for 3 trees.

For the fifth branch in the tree, Group I-III was selected for all 10 trees, fusion - was chosen for 7 of the trees, unfavorable site was chosen for 4 of the trees, tumor size > 5cm was chosen for 3 of the trees, and ≥ 1 and < 10 age was chosen for 2 of the trees.

For the sixth branch in the tree, Group I-III was selected for all 10 trees, fusion - was chosen for 7 of the trees, unfavorable site was chosen for 4 of the trees, tumor size > 5cm was chosen for 3 of the trees, and <1 and ≥ 10 age was chosen for 2 of the trees.

For the seventh branch in the tree, Group I-III was selected for all 10 trees and fusion + was chosen for 7 of the trees.

For the eight branch in the tree, Group IV was selected for all 10 trees, fusion – was chosen for 8 of the trees, and 1 metastatic site was selected for 2 of the trees.

For the ninth branch in the tree, Group IV was selected for all 10 trees, fusion – was chosen for 8 of the trees, and > 1 metastatic site was selected for 2 of the trees.

For the tenth branch in the tree, Group IV was selected for all 10 trees and fusion + was selected for 8 of the trees.
